# Complete genome sequence of *Hyphomicrobium sp*. strain 1Nfss2.1 from marine sediments of the Berre lagoon, France

**DOI:** 10.1128/mra.00254-25

**Published:** 2025-07-14

**Authors:** Manon Bartoli, Nathalie Pradel, Stéphanie Fouteau, Zoé Rouy, Corinne Cruaud, Thierry Nadalig, David Vallenet, Pedro H. Oliveira, Agnès Hirschler-Réa

**Affiliations:** 1 Aix Marseille Univ, Université de Toulon, CNRS, IRD, MIO 27017, Marseille, France; 2 Génomique Métabolique, Genoscope, Institut François Jacob, Commissariat à l’Energie Atomique (CEA), CNRS, Université Evry, Université Paris-Saclay 27048, Gif-sur-Yvette, France; 3 Genoscope, Institut François Jacob, CEA, Université Paris-Saclay 27048, Evry, France; 4 Génétique Moléculaire, Génomique, Microbiologie (GMGM), Université de Strasbourg, UMR 7156 CNRS 27083, Strasbourg, France; Montana State University, Bozeman, Montana, USA

**Keywords:** bacterial genome, *Hyphomicrobium*, chloromethane, nitrate

## Abstract

We report the complete genome sequence of *Hyphomicrobium sp*. strain 1Nfss2.1, isolated from coastal marine sediments of the Berre lagoon, France. The genome consists of a 4,097,693-bp circular chromosome. It contains the genes involved in the Cmu chloromethane degradation pathway.

## ANNOUNCEMENT

The Berre lagoon, gathering steel industries and oil refineries, is open onto the Mediterranean Sea (1). The genome sequence of a *Hyphomicrobium sp*. isolated from this environment provides a resource to understand the adaptation mechanisms of microorganisms to marine anthropogenically disturbed environments.

Isolation of strain 1Nfss2.1 was performed using the Hungate technique (2) from 5 mL of sediments collected with a piston. The medium contained 0.1 g.L^−1^ KH_2_PO_4_, 0.1 g.L^−1^ K_2_HPO_4_, 0.2 g.L^−1^ NH_4_Cl, 7 g.L^−1^ NaCl, 1.3 g.L^−1^ MgCl_2_.6H_2_O, 0.1 g.L^−1^ KCl, 0.05 g.L^−1^ CaCl_2_.2H_2_O, 0.1% trace elements solution (DSMZ 320), 0.1% Se-Ni-Mo-W solution (DSMZ 385), 1% Balch’s vitamins (3), 0.1 g.L^−1^ B12 vitamin, and 20 mM KNO_3,_ under N_2_ atm. Chloromethane was added at 4 mM, and pH was 7.5. Agar was added at 1.5% if needed. Three sub-cultures of 50 mL were performed at 30°C, without shaking, prior to serial dilutions on solid medium using roll tubes (4). Colonies developed after three weeks. From a colony, 10^−2^ serial dilutions were repeated in liquid medium until extinction. DNA was extracted using the Wizard Genomic DNA Purification Kit (Promega) from 5 mL of culture in the same medium.

Genome sequencing was carried out using Illumina and Nanopore Technologies. DNA was quantified using the Qubit 2.0 Fluorometer (Thermo Fisher). For Illumina, 250 ng DNA was sonicated to a mean size of 380 bp, using a Covaris E210, and profiles were visualized on an Agilent Bioanalyzer DNA chip. The fragments were end-repaired, 3′-adenylated, and ligated to NEXTflex barcodes (Bioo Scientific Corporation) (5). The ligated product was amplified by 12 PCR cycles using the KAPA HiFi Library Amplification kit (KAPABIOSYSTEMS) and purified with 0.6× AMPure XP (Beckman Coulter). The library was sequenced on an Illumina MiSeq with a MiSeq Reagent Kit-v2 (2 × 150 bp). A total of 1,313,850 paired-end reads were obtained. The Illumina reads were trimmed by removing low-quality nucleotides (Q < 20), adaptors, and sequences <30 nt, using fastx_toolkit-v0.0.14 (http://hannonlab.cshl.edu/fastx_toolkit).

Nanopore sequencing was performed according to Oxford Nanopore Technologies (Oxford, UK). The library was prepared with 400 ng DNA following the Native Barcoding Kit 24-v14 protocol, with the SQK-NBD114.24 ligation kit. The library was sequenced using an R10.4.1 flow cell and the GridION device with the MinKNOW-v6.0.14 and Guppy-v7.4.14+d47971cbb software. A total of 97,336 reads were obtained with an N50 of 18 Kb. Assembly was performed with Flye-v2.9.5 (6) on long reads filtered with Filtlong-v0.2.1 (min length 6,000; keep_percent 90; https://github.com/rrwick/Filtlong), resulting in an assembly in a single loop-edge representing a circular chromosome with a 102× mean coverage. Polishing was done with long reads using Medaka-v2.0.1 (https://github.com/nanoporetech/medaka), and with Pypolca-v0.3.1 (7). Completeness (99.20%) and contamination (0.89%) were estimated using CheckM (8). Default settings were used for all software. Annotation was performed at the Microscope platform-v3.17.3 (9).

The whole genome consisted of a 4,097,693 bp circular chromosome with an average G+C content of 62.95%. A total of 4,187 coding DNA sequences were predicted. NCBI blastn analysis indicated 97.9% identity over 98% length of the 16S rDNA sequence of *Hyphomicrobium album* XQ2^T^, isolated from soil, in China (10). The average nucleotide identity value between the two genomes was only 77.6%, suggesting that strain 1Nfss2.1 may represent a new species (EZ BioCloud ANI Calculator, 21 April 2025). It is the first genome of *Hyphomicrobium sp*. that possesses both genes of the chloromethane degradation pathway and genes for the complete anaerobic nitrate respiratory chain.

## Data Availability

This whole genome project and the raw data have been deposited in ENA under the numbers PRJEB86990 and ERA32774194, respectively. The chromosome sequence is available at ENA under the accession no. OZ251603.
